# History, development, and current status of food safety systems worldwide

**DOI:** 10.1093/af/vfy016

**Published:** 2018-08-30

**Authors:** Margaret D Weinroth, Aeriel D Belk, Keith E Belk

**Affiliations:** Department of Animal Sciences, Colorado State University, Campus Delivery, Fort Collins, CO

**Keywords:** food safety, Global Food Safety Initiative, Hazard Analysis Critical Control Point

**Implications**

- Hazard Analysis Critical Control Point developed and remains the principal management method for reducing risk of foodborne illness.
- In developed countries, food safety has begun to cast a wider net and incorporate intentional food adulteration, food fraud, and sustainability.
- In developing countries, barriers to effective food safety systems include costs, a lack of surveillance programs, and limited opportunities for employee education.

## Introduction

Immediately following the 1993 Jack-in-the-Box outbreak caused by *Escherichia coli* O157:H7, the United States began to look for a more robust regulatory food safety system than previously employed. In the same time frame in the United Kingdom, an outbreak of Bovine Spongiform Encephalopathy (**BSE**) eroded public trust in the food safety systems of Western Europe. As a result, there was increased interest in implementing the Hazard Analysis Critical Control Point (**HACCP**) system worldwide. Although the ideas incorporated into HACCP principles were developed decades earlier, the 1990s saw a focus on implementation of the system throughout developed food production systems based on the National Advisory Committee on Microbiological Criteria for Foods (**NACMCF**)’s seven principles which were subsequently mostly adapted by Codex Alimentarious. During this period of time, there were increased governments and private companies that required HACCP implementation. In the United States alone, HACCP was estimated to reduce foodborne illness by 20% during the 7 yr after its implementation. During HACCP adoption, many food retail and foodservice purchasers also developed additional unique specifications that suppliers had to adhere to in order to market their products, the idea being to improve consumer confidence in food safety management systems. This resulted in creation of the Global Food Safety Initiative (**GFSI**) system for benchmarking additional voluntary food safety management standards against preferred methods for reducing foodborne illnesses (first in Europe, and later adopted in the United States and globally) which reduced redundancy and helped us to bring global consistency to food safety. Although developed countries now mostly all adhere to core HACCP principles of food safety, ideas dealing with traceability, vulnerability to food fraud, and intentional adulteration are now being considered to further bolster food safety. On the other hand, developing countries appear to have had varied success in implementing similar food safety management systems, and many countries still struggle with high numbers of foodborne illnesses.

At the broadest level, the HACCP system is a preventive-based method for assuring food product safety. Biological, physical, and chemical hazards can be prevented, reduced, or eliminated through this system. In addition to the management of hazards, record keeping demonstrating adherence to HACCP is included in the system.

## HACCP Formation and Acceptance

### Early history

**Figure 1.**
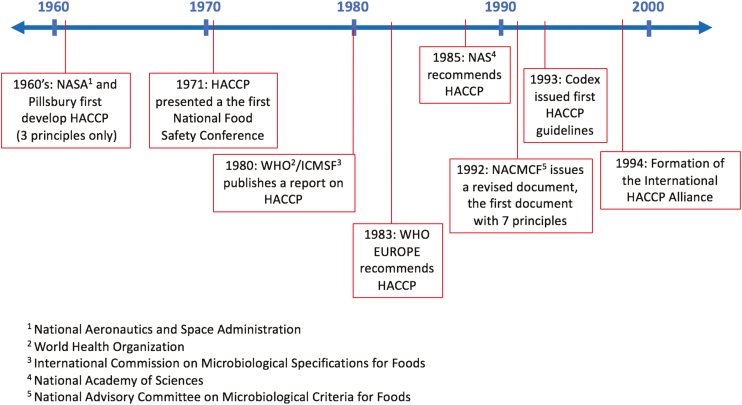
Timeline of major events leading to the creation of HACCP.

Figure 1 shows a timeline of the major events leading to the development of HACCP principles. Prior to the creation and adaption of the HACCP system, the precursor to quality control was Total Quality Management (**TQM**). The TQM system was first introduced by W. Edward Deming and relied on the concept of continuous improvement (Deming, 1986). The HACCP concept was first developed in the 1960s by the U.S. National Aeronautics and Space Administration (**NASA**), working with Pillsbury, to ensure crumb- and pathogen-free food that had extensive shelf-life properties for space travel—the first pathogen monitoring and measurement requirement imposed on the food industry (Lachance, 1997). The program was formed, with modification, based on the U.S. Army Natick Research, Development, and Engineering Center’s methods of ensuring quality medical supplies (Cronk, 1994). However, the NASA HACCP program imposed strict pathogen limits on food and required cumbersome testing procedures, which let little of the food produced pass pathogen control. As a result, this system required modification in order to be practical for commercial food production. To assist with this goal, NASA requested help from food industry companies, including Pillsbury, who first developed the HACCP approach (Cronk, 1994). From the NASA standpoint, Dr. Paul Lachance led the food safety of flight food and Dr. Howard Bauman led the Pillsbury team (Lachance, 1997). The implementation of this program allowed for the reduction of risk related to foodborne pathogens in food, although the original HACCP plan only consisted of three principles as opposed to the seven we know today. As Pillsbury became engaged in improving the space programs’ HACCP system, they also began to implement it into their own food safety practices within the company. In the spring of 1971, HACCP was presented to the food industry for the first time at the first National Conference on Food Protection (Ross-Nazzal, 2007). The following year, Pillsbury began teaching HACCP classes to Food and Drug Administration (**FDA**) inspectors and HACCP was implemented in low-acid canning regulation (Cronk, 1994).

**Figure 2.**
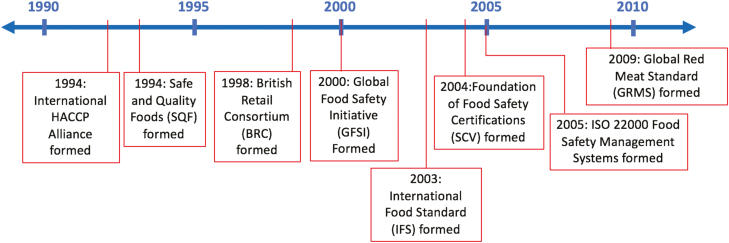
Timeline of introduction of food safety management systems.

Following a 1980 World Health Organization (**WHO**)/International Commission on Microbiological Safety of Foods (**ICMSF**) report on HACCP, WHO EUROPE recommended its use in 1983 (Huss et al., 2003). In 1985, the National Academy of Science concluded that HACCP, as opposed to another common idea of random testing of foods, was an adequate method of ensuring wholesome food (National Research Council Subcommittee on Microbiological Criteria, 1985). Another outcome from the 1985 report was formation of the NACMCF, who further encouraged adoption of HACCP through the development and publication of resources based on education and implementation of the idea. The 1992 revision of this document from NACMCF presented the seven core principles central to HACCP for the first time. During this time, HACCP found support in many food safety meetings and groups, including the 1993 adoption of the *Guidelines for the Application of the Hazard Analysis Critical Control Point System* by the Codex Alimentarius Commission (an international intergovernmental body in the Joint Food Standards Program established by the Food and Agriculture Organization of the United Nations [FAO] and WHO). A timeline of the introduction of food safety management systems is shown in Figure 2.

### 1993 outbreak and U.S. aftermath

In the early 1990s, there was talk of HACCP as an effective tool to control food safety hazards in some circles, but there was still little change occurring in the meat and poultry industry (Ross-Nazzal, 2007). The lack of impetus to move to a HACCP-based food safety management system changed with the *E. coli* O157:H7 outbreak that was linked to undercooked beef patties from the Jack-in-the-Box fast food chain in 1993; an outbreak resulting in the death of four children and the sickening of over 700 people across multiple states (Seo et al., 2014). This high-profile outbreak ignited a national conversation about current food safety regulation in the United States. In response to this outbreak, Jack-in-the-Box hired food safety expert Dr. David Theno and implemented HACCP, becoming the first fast food company in the world to do so (Ross-Nazzal, 2007). One of the USDA Food Safety and Inspection Service responses to the outbreak was to propose the Pathogen Reduction/HACCP Systems rule in 1995 that was finalized as a regulation in 1996 (U.S. 9 CFR Part 304). By 2003 and following several years of implementation time, the USDA Economic Research Service estimated that use of HACCP systems had reduced foodborne illness by 20% in the United States (Ollinger and Mueller, 2003).

## Formation of the International HACCP Alliance

As HACCP implementation became more widespread and evolved, many stakeholders across the meat and poultry industries saw need for a more unified understanding of the system. To meet this need, the International HACCP Alliance (**IHA**) was formed and based at Texas A&M University, even before HACCP became a regulatory requirement (Jackson et al., 1996). The purpose of the alliance was (and remains to this day) to provide a uniform program for safer meat and poultry products and to bring together industry associations, educational foundations, professional organizations, university experts, government cooperators (both within the United States and internationally), and third-party private companies. Originally targeting a three pronged approach: 1) the alliance provides an avenue for training and research, 2) it makes available incentives and resources for early adoption, and 3) it serves as a unified communicator of HACCP’s role in food safety through food production (Jackson et al., 1996). Today, the IHA serves as the international authority on HACCP systems and regulatory compliance, and provides curricula and accreditation for HACCP classes while facilitating an understanding of HACCP philosophies through cooperation with its partners.

## Tantum International Standard Development of Food Safety With HACCP

Although the United States was reevaluating their food safety systems as a result of high profile outbreaks, including the Jack-in-the-Box outbreak, the United Kingdom was forced to revisit their food safety regulations due to lost credibility and consumer confidence resulting from regulatory decisions that were made concerning BSE in the late 1980s and early 1990s (Millstone and Van Zwanenberg, 2003). Around this same time, in 1989, the Richmond Report was published in the United Kingdom, recommending adoption of HACCP throughout the food industry (Demortain, 2007). Council Directive 94/43/EEC (1994) built on previous directives in the E.U. (including the 1991 directive that placed the responsibility of product safety on the industry and the 1993 directive on hygiene of foodstuffs) to detail the rules and application of the HACCP system (Huss et al., 2003).

During this time frame, and in parallel, both of the voluntary standards known as Safe Quality Foods (**SQF**) program (1994) and the British Retail Consortium (**BRC**) in England (1998) were developed, eventually becoming “benchmarked” by the GFSI (Robinson, 1999; British Retail Consortium, 2018). Other countries/companies also began to implement their own food safety audits to improve food safety. For example, the International Food Safety Standard (2003) implemented by Germany and France and the 2004 Foundation of Food Safety Certification (**SCV**) in the Netherlands both used HACCP to reduce or eliminate hazards; both also were eventually components of benchmarked GFSI standards. In all cases, implementation with these standards was expected to be audited by third-party certification bodies.

As more companies and countries worldwide implemented differing food safety programs and standards, a single company with an abundance of customers in an international market could be made to comply with many different standards each year. As a result, there was a need to cross-standardize among such requirements and criteria. In addition, and particularly in Europe as a consequence of the BSE scandal, the result was formation of GFSI in 2000. This new platform devised methods for “benchmarking” the components of differing “schemes” (unique food safety management standards) based on existing philosophies to establish credibility among the schemes such that they would be acceptable to retailers regardless of which scheme was used by a supplier (Crandall et al., 2012). Through the GFSI system, many previously implemented food safety standards were benchmarked by GFSI, including those mentioned previously like SQF and BRC. In 2008, H-E-B and Walmart were the first regional and national grocers, respectively, to adopt Global Food Safety Initiative (**GSFI**) as a requirement in their supply chain in the United States, leading to adoption of the GFSI concept by their suppliers as a requirement for doing business (Crandall et al., 2012). As of 2018, there are 14 GFSI-Recognized Certification Programmes that meet benchmarking requirements, in addition to China’s HACCP program that is acknowledged for its equivalence to GFSI technical requirements (GFSI, 2018). All GFSI benchmarked standards require the implementation of HACCP systems.

## Current International Standards

**Figure 3.**
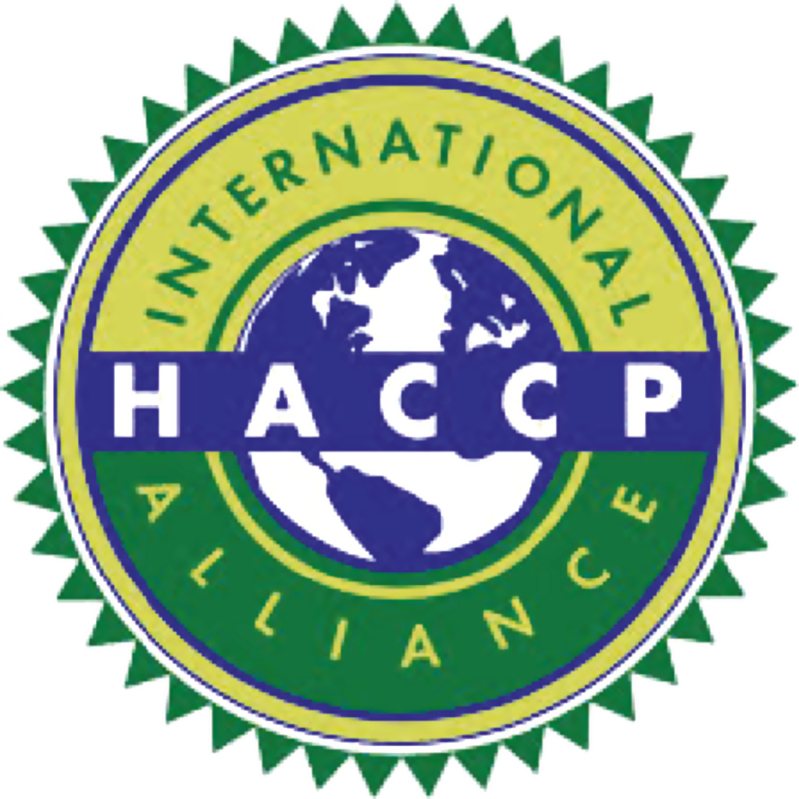
International HACCP logo.

Although HACCP has been internationally recognized since the mid 1990s, there are many certifications and systems that integrate and build upon the HACCP approach. Figure 3 shows the international HACCP logo that is recognized as conveying the HACCP principles. As already mentioned, GFSI recognized, via their benchmarking program, several international standards that incorporate HACCP. Others range from country to country and from company to company as to the specific programs implemented.

### International Organization for Standardization certification

Another key international standard is ISO 22000, a standard developed by the International Organization for Standardization (**ISO**) that is specific to food safety, but that is based off of the ISO 9000 family of quality management systems standards. Before ISO 22000, companies could implement ISO 9000 in conjunction with HACCP. The ISO 9000, though, is not specific to food safety, but instead is a quality management system that allows companies to ensure that they meet quality specifications in business transactions (Efstratiadis et al., 2009). The ISO 22000 standard is a food safety-specific standard that integrates the HACCP system as described by Codex Alimentarius, along with three other elements: interactive communication, prerequisite programs, and system management. The ISO 22000 family of food safety management standards is made up of several standards, such as ISO/TC 22002 prerequisite programs and 22005, traceability in the food chain (ISO, 2018). An organization can be certified (or certificated) in specific management standards by a third-party auditor to demonstrate compliance, but ISO is, in itself, not a certification body—it serves as an international standards development body like Codex and other such bodies.

**Figure 4.**
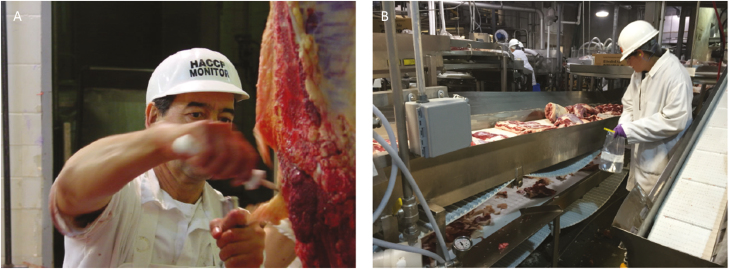
Inspection of a carcass (A) and meat products (B) in a meat processing facility.

Today, the 164 member countries of the World Trade Organization (**WTO**) recognize the CAC standards as food safety policy that meets international expectations for food safety management. These standards are set forth in the Joint FAO/WHO Food Safety Standard Programme *Recommended International Code of Practice. General Principles of Food Hygiene* which highlights a HACCP approach to food safety (Codex Alimentarius Commission, 2001). Figure 4 shows inspection of a carcass and meat products in a processing facility.

## Food Safety Systems of Developed Countries

Throughout the globalization of food safety, many developed countries have implemented robust regulatory food safety systems. Although the first priority of these systems was to reduce risk to human health associated with specific foods through reduction and elimination of hazards, several broader goals have emerged in these countries in the last few years. Focus areas of an expanding food safety view are management of hazard risk throughout the farm to fork continuum, implementation of suitable practices and traceability, addressing food terrorism and intentional adulteration, vulnerability to food fraud, and the rise of antibiotic resistance. Australia and New Zealand, Canada, the United States, and the E.U. have all refined their food safety practices in recent years.

### Australia and New Zealand

Australia and New Zealand have always worked closely with each other in terms of food production and trade. The first joint organization between the two countries in terms of a food system was established in 1991 and is today known as Food Standards Australia New Zealand (**FSANZ**). In 1996, a treaty between the two countries was signed to allow, among other benefits, a joint food standard system (Australian Government Department of Health, 2018). It was also in 1996 that FSANZ endorsed the Codex Alimentarius Commission guidelines for HACCP. Although the formal endorsement for HACCP came in the mid-1990s, Australia was much more proactive in HACCP implementation across food sectors and had a much more industry-driven implementation strategy than other countries in the 1980s (Ropkins and Beck, 2000), probably due to the great amounts of food that were exported. The FSANZ agency is responsible for food standards from farm to fork and is enforced by local government (Jol et al., 2012). One of the challenges associated with food production, particularly in Australia, is the fragmented enforcement of food standards that has been pointed out to not be as linear was other systems, such as the E.U.s (Ghosh, 2014).

### Canada

In Canada, the Canadian Food Inspection Agency (**CFIA**) is charged with ensuring that meat and poultry products leaving federally inspected plants, or that are imported, are safe. Canada has a long history of food safety regulation and was the first to implement a system based on HACCP principles, the system known as the Quality Management Program (**QMP**) in 1992 (Canadian Food Inspection Agency Government of Canada, 2012). Although HACCP itself was recognized and encouraged as a method to reduce foodborne illness, it did not become mandatory until 2005 in federally registered meat and poultry establishments (Canadian Food Inspection Agency Government of Canada, 2012).

### The United States

The United States has fully embraced HACCP as a regulatory requirement in meat and poultry production since implementation of the Pathogen Reduction, HACCP Systems Final Rule in 1996. Although not directly tied to meat and poultry slaughter and processing, the 2011 charge to FDA by Congress in the Food Safety Modernization Act (**FSMA**) further demonstrated a conclusive shift in the U.S. government’s attitude about food safety. Although the FSMA does not affect meat and poultry (which are regulated specifically by USDA-FSIS), this legislation still has important implications to animal protein producers that generate multiple-ingredient products or items destined to pet food production. The biggest shift in FDA regulations, compared with previous requirements for foods that are regulated by FDA, was the change in focus to prevention measures as opposed to reaction to positive tests (Grover et al., 2016). Specific pieces of FDA Preventive Controls regulations include the ability to trace products and include mitigation strategies for intentional adulteration (Nakuja et al., 2015).

### The European Union

The E.U. has proactively adopted food laws for its 28 member countries that are applicable to other countries (i.e., third countries) that trade with member nations to the E.U. The European Food Safety Authority (**EFSA**) was established by the General Food Law in 2002 and is responsible for risk assessment (European Food Safety Authority, 2018). Another more recent focus of the E.U. has been the move towards more sustainable food resources. Although this includes issues associated with facets outside of food safety, the 2017 European Environment Agency report “Food in a green light - A systems approach to sustainable food” also highlights a desire to phase out use of harmful chemicals (such as pesticides of concern) throughout the food chain (European Environment Agency, 2017).

## Emerging Markets and Developing Countries (South America, Pacific Rim)

Development and adoption of food safety systems is very inconsistent among developing countries. In such developing countries, where economies are still endeavoring to increase in robustness, there are several barriers to successfully implementing HACCP or other food safety systems. Some countries have required partial adoption of HACCP in their plants, whereas others have struggled. In 2009, only one-third of poultry plants included in a survey in Mexico were HACCP certified by a third party (Maldonado et al., 2005). When those plants that implemented HACCP were asked what the main hurdles were towards implementation, the cost of equipment was considered the greatest barrier, followed by costs related to external consultants that they felt were necessary to assist with preparation of documentation, with the least important consideration being staff training (Maldonado et al., 2005).

As reported in a 2006 study of Turkey, which is a member of the Food and Agriculture Organization and CAC, there was little adoption of HACCP or even perquisite programs. In a survey of over 100 food businesses in Ankara, Turkey, only 7% reported HACCP implementation (Baş et al., 2006). This latter study found that a major problem surrounding implementation is that food workers often lack interest in learning about food safety programs (Baş et al., 2006). Another need that was identified was that because businesses have a limited understanding of implementation, regulatory authorities need to clarify goals and ensure uniform application of the principles. With regard to the implementation of HACCP in developing countries, Ropkins and Beck (2000) identified that education and training, as well as availability of information concerning what hazards are likely to occur in the region, were major hurdles to HACCP implementation. As countries continue to develop, it is likely that more will require HACCP, such as Russia’s 2014 mandate of the system (Chernukha et al., 2015). Three specific countries of interest, due to their opportunities for export growth as emerging markets, include China and Latin America.

### China

Red meat production in China has grown at a rate of 5.8% annually, with food safety being heavily scrutinized by the public (Zhou et al., 2012). However, although China is a major producer of meat internationally, less than 10% of their production facilities are HACCP certified. And, beyond this concern, issues regarding antibiotic residues, microbial contamination, and use of illegal drugs are also of concern (Zhou et al., 2012). Although there are concerns about Chinese meat production, there are efforts to increase the safety of meat products. Research expenditures towards improving food safety, while still behind other countries, has increased in China. Additionally, the government of China has introduced the China HACCP program that GFSI recognized as technically equivalent to meeting its guidance documents (Table 1).

**Table 1.** List of acronyms used throughout the article

Acronym	Definition
HACCP	Hazard Analysis Critical Control Point
GFSI	Global Food Safety Initiative
WHO	World Health Organization
ICMSF	International Commission on Microbiological Safety of Foods
NACMCF	National Advisory Committee on Microbiological Criteria for Food
CAC	Codex Alimentarius Commission
FAO	Food and Agriculture Organization of the United Nations
IHA	International HACCP Alliance
SQF	Safe Quality Foods
BRC	British Retail Consortium
SCV	Foundation of Food Safety Certification
WTO	World Trade Organization
FSANZ	Food Standards Australia New Zealand
CFIA	Canadian Food Inspection Agency
QMP	Quality Management Program
FSMA	Food Safety Modernization Act
EFSA	European Food Safety Authority

### Latin America

Limited formal reporting of foodborne illness (i.e., limited surveillance) also limits understanding of the full scope of food safety outbreaks that may occur in Latin America. Nonetheless, it was estimated that 6000 outbreaks occurred due to several pathogens between 1993 and 2002. In several instances, multiple Latin American countries have been banned from exporting due to outbreaks of different pathogens (Akhtar et al., 2014). The implementation of food safety systems in these countries is inconsistent and variable. For example, Mexico has legislation regarding HACCP (NOM‐251‐SSA1‐2009), though some food safety laws are still voluntary and adopted more widely by tourism centers or exporters, rather than uniformly across the country to increase safety of meat products (Leon and Paz, 2013). On the other hand, Brazil (the largest exporter of beef in the world) has implemented mandatory inspection in all meat and poultry facilities for production, processing, and distribution (Carneiro and Kaneene, 2017).

## Conclusion

Development of HACCP over the past 50 yr has led to documented improvement in food safety outcomes (Ollinger and Mueller, 2003). Throughout the evolution of HACCP, there was refinement from the original idea to the seven principles in practice today. In terms of industry cooperation, GFSI has allowed multiple food safety management systems to be benchmarked and considered acceptable for use; all require HACCP. Today, food safety systems differ between developed and developing countries. Developed countries all have the core components of reducing foodborne illness, with additional considerations that include traceability, sustainability, food fraud, or food defense. On the other hand, developing countries still struggle with uniform regulatory implementation of food safety standards. To continue to decrease foodborne illness worldwide, focus needs to be expended on increasing implementation of these proven systems in developing countries.

## About the Authors

Margaret (Maggie) Weinroth is a Ph.D. Candidate in the Animal Sciences Department at Colorado State University, where she also earned her B.S. and M.S. in Animal Sciences. Her current work focuses on applying next-generation sequencing technologies (mainly shotgun metagenomics and 16S) to food safety concerns; such as the dissemination of antibiotic resistance genes and virulence factors through food production systems. Previously, she evaluated how different antimicrobial treatment strategies affect the bovine resistome.

Aeriel Belk is a doctoral student in Animal Sciences at Colorado State University. Her primary research focus is investigating the decomposition-associated microbiome. Specifically, she is modeling the postmortem succession of microbes using next generation sequencing to better understand the shelf-life of meat and poultry products. She obtained a Bachelor of Science in Animal Sciences from Colorado State University and a Master of Science with certificates in meat science and food safety from Texas A&M University.

Dr. Keith Belk is a Professor and holds the Ken & Myra Monfort Chair in the Center for Meat Safety & Quality, Department of Animal Sciences at Colorado State University. He also is an adjunct Professor in the Colorado School of Public Health and has served as the consulting Director of Scientific Affairs for Food Safety Net Services, LLC since 2008. He has been employed by Safeway, Inc. as a beef buyer, and by the USDA-AMS in Washington, DC, as an International Marketing Specialist.
